# Supplementary figures and images for: Bioremediation of engine-oil contaminated soil using local residual organic matter
Source: PeerJ. 2019 Aug 1;7:e7389. doi: 10.7717/peerj.7389 (PMC6679911; doi:10.7717/peerj.7389)

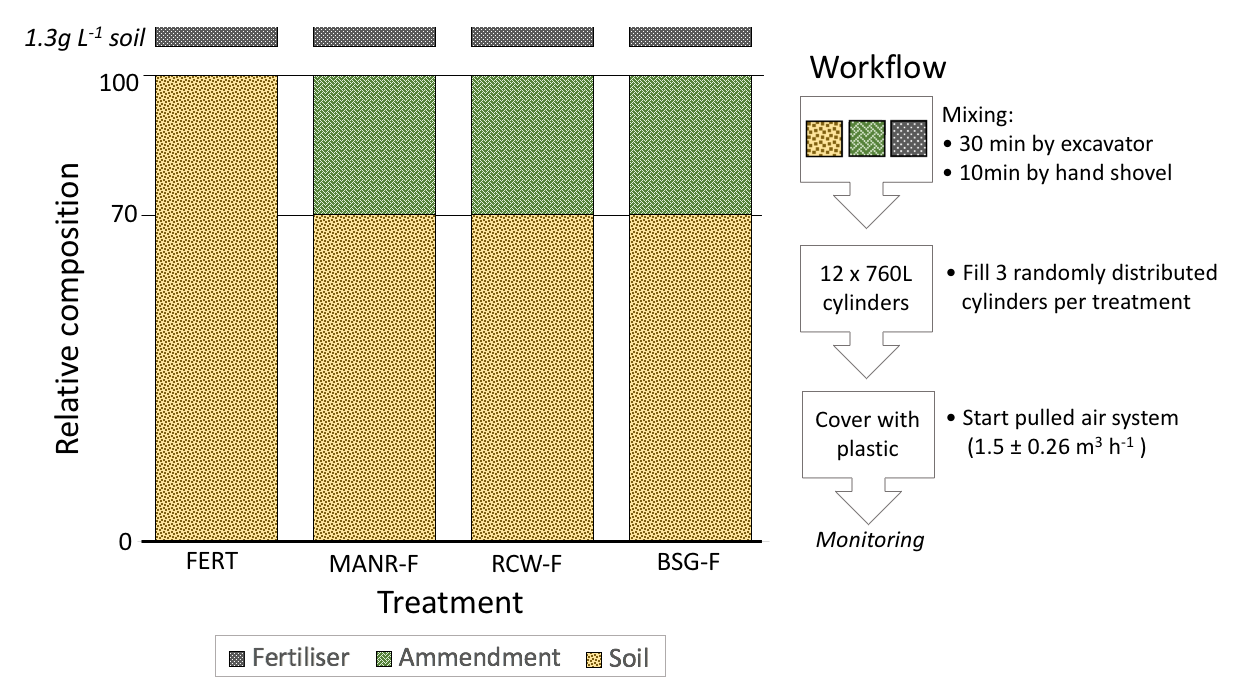

Supplement: Supplemental Information 1 [file peerj-07-7389-s001.png]
